# Evaluation framework for healthcare integration pilots in the Basque Country/Marco evaluativo de las experiencias de integración asistencial en el País Vasco

**Published:** 2012-05-29

**Authors:** Nuria Toro, Maite Paino, Iñaki Fraile, Ricardo Samper

**Affiliations:** Senior Researcher, O+berri (Basque Institute for Healthcare Innovation), Sondika, Basque Country, Spain; Deputy Director of Human Resources, Basque Health Service-Osakidetza, Vitoria-Gasteiz, Basque Country, Spain; Officer of the Subdepartment of Healthcare, Basque Health Service-Osakidetza, Vitoria-Gasteiz, Basque Country, Spain; Pharmacist of the Subdepartment of Healthcare, Basque Health Service-Osakidetza, Vitoria-Gasteiz, Basque Country, Spain

**Keywords:** healthcare integration, assessment, outcomes, evaluation framework, integración asistencial, evaluación, resultados, marco evaluativo

## Introduction

The Basque Health Service is currently running a series of pilots in healthcare integration with the objective of obtaining better outcomes in the management of chronic patients, through an improvement in the quality of care and in resource use efficiency. Despite evidence that the effectiveness of specific interventions in the management of chronic patients, there is no sound evidence to assess the extent to which models and policies on health integration contribute to improvements in quality of care and cost reduction within health systems. In this context, it is essential to evaluate healthcare integration pilots to determine to what extent expected outcomes are achieved in the medium to long term and, therefore, how integration may contribute to the sustainability of health systems.

## Description

**Figure 1.**
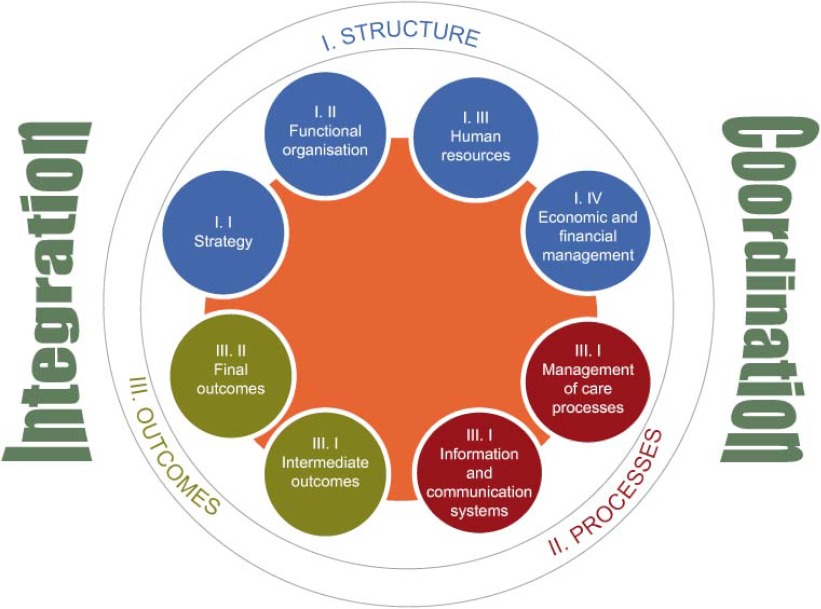
Schematic diagram of the evaluation framework. **Structure element:** this has been defined as the type of structure on which the new organisational model is based. It has four dimensions: strategy, functional organisation, human resources and economic and financial management. **Process element:** this is a measure of the degree of implementation of healthcare processes and integrated information and communication systems. It has two dimensions: management of care processes and information and communication systems. **Outcome element:** this is a measure of how effective the integration/coordination model is in achieving the objectives set and is without any doubt the most important element of the evaluation. It has two dimensions: intermediate outcomes, which are instrumental variables, positive in themselves and a step towards achieving the final outcomes; and final outcomes which measure the direct impact on patients, professionals and the health system.

The objective is to establish an evaluation framework to assess the healthcare integration pilots in the Basque Country that will make it possible to compare results at regional, national and international levels. The evaluation has structure, process and outcome elements (see Figure 1).

### Methods

A series of indicators (see Table 1) for each of the dimensions were selected, on the basis of the international literature and clinical guidelines, by a group of multidisciplinary experts including primary and specialised care professionals (clinicians and managers), psychologists, pharmacists, economists and epidemiologists, among others.

### Development

**Figure 2.**
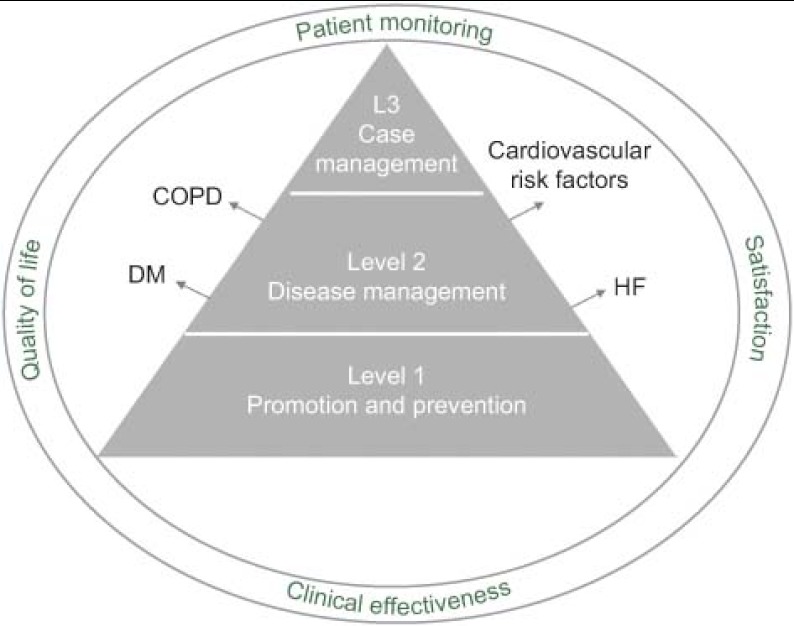
Population perspective for outcome indicators.

We have defined outcome indicators from a population perspective, bearing in mind that there is no single type of patient. Indeed, on the contrary, there is a wide range of types of patients with varying clinical risk and complexity. To consider this, we have stratified the population into three levels, **identifying ad-hoc indicators for each level** based on variables related to monitoring of patients, clinical effectiveness and quality of life (see Figure 2). Level 1 represents the general population for whom the focus is on promotion and prevention activities, while at Level 2 we need to consider disease management. Specifically, for this middle level, four medical conditions have been identified that, according to the literature, can benefit from an integrated approach to healthcare: diabetes mellitus (DM), chronic obstructive pulmonary disease (COPD), and heart failure (HF), as well as the presence of cardiovascular risk factors. Finally, Level 3 corresponds to patients with multiple diseases. In assessing patient satisfaction, the segment of the population a patient is in will be taken into account.

Both the indicator of “interprofessional collaboration” and those for “patient satisfaction” and “professional satisfaction” will be measured using questionnaires designed for the purpose. For the first of these, a questionnaire has been developed based on the D’amour model of interprofessional collaboration [1], with the collaboration of the original author. As for patient and professional satisfaction, we are currently translating and adapting (following the methodology recommended by WHO) the questionnaires developed by RAND, Ernst and Young, the Nuffield Trust and the English Department of Health to evaluate the integrated care pilots in England [2].

All the indicators of the “structure” and “process” elements have a scale ranging from 0 to 3: “degree of development: none-low-medium-high” while for “outcomes”, the scale also ranges from 0 to 3: 0 meaning that the situation worsens, 1 that there has been no change, 2 that the situation has improved but objectives have not been met and 3 that objectives have been met or exceeded compared to the previous period.

In March 2011, we started to assess the baseline situation of the integration pilots. From now on, we will carry out annual evaluations of the pilots, to analyse outcomes over the following years.

## Discussion and conclusions

Despite some international evidence that healthcare integration may improve the performance of systems, and patient quality of life and satisfaction, it is not clear that it will reduce costs (at least in the short term) or that any single combination of structures and methods will lead to optimal outcomes [3]. Given the fact that it is not possible to predict the outcomes of healthcare integration pilots, it is important that there is an evaluation process considering the various different aspects of integration. Accordingly, we have prepared an evaluation framework for the integration pilots running in the Basque Country, and this may be applicable to the evaluation of healthcare integration initiatives in other regions in Spain and beyond.

## Poster abstract Spanish

## Introducción

Hoy en día, el Sistema Sanitario Vasco está desarrollando una serie de experiencias en el ámbito de la integración asistencial con el objetivo de conseguir mejores resultados en la gestión del paciente crónico, a través de la mejora de la calidad de la atención y de un uso más eficiente de los recursos. A pesar de que existe evidencia de la efectividad de intervenciones específicas en la gestión del paciente crónico, no hay estudios sólidos que determinen hasta qué punto los modelos y las políticas de integración asistencial pueden contribuir a la mejora de la calidad y la reducción de costes en el sistema sanitario. En este contexto, la evaluación de las experiencias de integración asistencial es clave de cara a mostrar en qué medida los resultados esperados se consiguen a medio y largo plazo y por tanto, la integración puede contribuir a la sostenibilidad del sistema sanitario.

## Descripción

**Figura 3.**
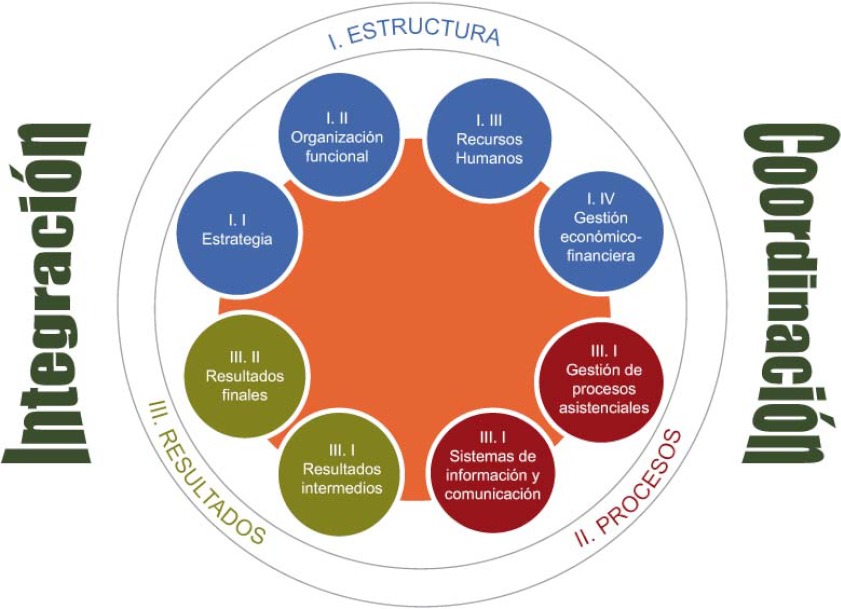
Esquema del marco evaluativo. **Eje Estructura:** se ha definido como “tipo de estructura sobre la que se levanta el nuevo modelo de organización”. Contiene 4 dimensiones: estrategia, organización funcional, recursos humanos y gestión económico-financiera. **Eje Procesos:** mide el grado de implementación de los procesos asistenciales y de los sistemas de información y comunicación conjuntos. Contiene 2 dimensiones: gestión de procesos asistenciales; y sistemas de información y comunicación. **Eje Resultados:** mide la eficacia del modelo de integración/coordinación en la consecución de sus objetivos y constituye sin duda el eje más importante de evaluación. Aquí se distinguen dos dimensiones. Por una parte, resultados intermedios, que son variables instrumentales, deseables en sí mismas y que contribuyen a la consecución de resultados finales. Y por otra, los resultados finales, que miden el impacto directo en pacientes, profesionales y el sistema sanitario.

El objetivo es evaluar las experiencias de integración asistencial desarrolladas en el País Vasco a través de un marco evaluativo que permita la comparativa en términos regionales, nacionales e internacionales. Las áreas de evaluación son: estructura, procesos y resultados (ver Figura 3).

### Método

Se han desarrollado una serie de indicadores (ver Tabla 2) para cada una de las dimensiones planteadas por un grupo de expertos multidisciplinar compuesto por profesionales de atención primaria y especializada (clínicos y gestores), psicólogos, farmacéuticos, economistas y epidemiólogos, entre otros, sobre la base de una revisión de literatura internacional y de guías de práctica clínica.

### Desarrollo

**Figura 4.**
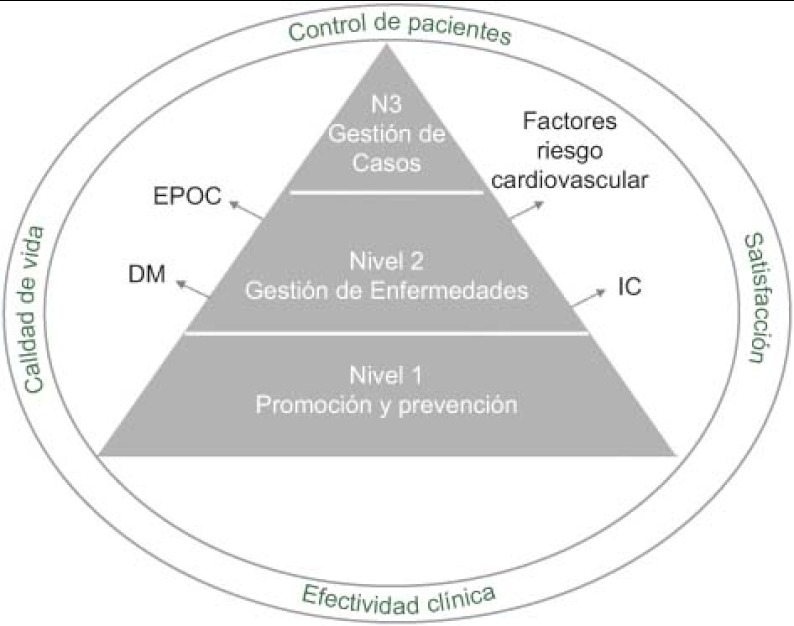
Perspectiva poblacional en los indicadores de resultados.

A la hora de definir los indicadores de resultados, se ha hecho desde una perspectiva poblacional, teniendo en cuenta que el paciente no es único sino que existen distintos grupos de pacientes en función de su riesgo y complejidad. Así, se ha estratificado a la población en 3 niveles, **identificándose indicadores ad-hoc para cada estrato** en las variables control del paciente, efectividad clínica y calidad de vida (ver Figura 4). En el Nivel 1, están las actividades de promoción y prevención, en el Nivel 2, “gestión de enfermedades”, se han identificado 4 condiciones o patologías que según la literatura, van a beneficiarse de un enfoque de integración asistencial: DM, EPOC, IC y factores de riesgo cardiovascular. Por último, en el Nivel 3 están los pacientes pluripatológicos. La satisfacción también tendrá en cuenta el segmento de población al que pertenece el paciente.

Tanto el indicador de “colaboración interprofesional” como los indicadores de “satisfacción de pacientes” y “satisfacción de profesionales” se van a medir a través de cuestionarios diseñados al efecto. En relación al primero, se ha desarrollado un cuestionario derivado del “Modelo D´Amour de Colaboración Interprofesional” [1], con participación de la propia autora. En cuanto a la evaluación de la satisfacción de pacientes y profesionales, se está llevando a cabo la traducción y adaptación (acorde al método fijado por OMS) de los cuestionarios desarrollados por RAND, Ernst & Young, Nuffield Trust y el Departamento de Sanidad inglés para la evaluación de las experiencias de integración inglesas (“Integrated Care Pilots”) [2].

En relación a la escala, todos los indicadores de los ejes “estructura” y “procesos” tienen una posible puntuación que va de 0 a 3 “grado de desarrollo nulo-bajo-medio-alto” y para “resultados”, la puntuación también va de 0 a 3 y en este caso 0, si empeora la situación, 1 si se mantiene la situación, 2 si mejora la situación, sin cumplir objetivos, y 3 para el caso de que cumpla o exceda los objetivos marcados con relación al periodo anterior.

En Marzo del año 2011 se ha iniciado el análisis de la situación basal de las experiencias de integración. A partir de ahí, se procederá a la evaluación anual de las mismas y de esta forma se podrán analizar sus resultados en los años subsiguientes.

## Discusión y conclusiones

A pesar de que la evidencia internacional disponible afirme que la integración asistencial puede mejorar el desempeño del sistema, la calidad de vida y la satisfacción de los pacientes, no es tan claro que reduzca costes (al menos en el corto plazo) y tampoco existe una combinación única de estructuras y métodos que generen resultados óptimos [3]. Dado que no se pueden anticipar cuáles serán los resultados de las experiencias de integración asistencial, se necesita llevar a cabo un proceso de evaluación que integre los diferentes aspectos involucrados en la integración. Por ello, se ha elaborado un marco evaluativo para las experiencias de integración asistencial desarrolladas en Euskadi, cuyo uso puede extenderse a la evaluación de iniciativas de integración del resto de regiones y Comunidades Autónomas.

## Figures and Tables

**Table 1.**
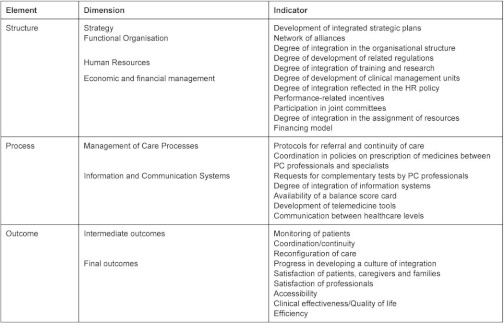
List of evaluation framework indicators by element and dimension

**Table 2.**
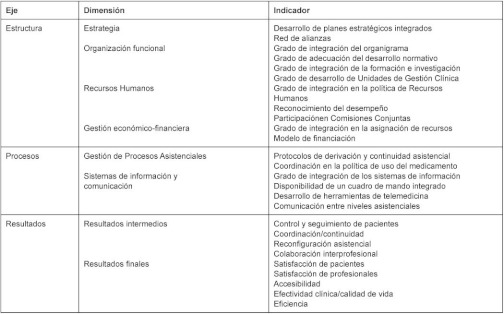
Relación de indicadores del marco evaluativo por eje y dimensión
